# Does contrast echocardiography induce increases in markers of myocardial necrosis, inflammation and oxidative stress suggesting myocardial injury?

**DOI:** 10.1186/1476-7120-3-21

**Published:** 2005-08-17

**Authors:** Fabian Knebel, Ingolf Schimke, Stephan Eddicks, Torsten Walde, Reinhard Ziebig, Sebastian Schattke, Gert Baumann, Adrian Constantin Borges

**Affiliations:** 1 Universitätsmedizin Berlin, Medical Clinic for Cardiology, Angiology, Pulmology, Charité Campus Mitte, Germany; 2 Paritätisches Krankenhaus Lichtenberg, Fanningerstraße 32, 10365 Berlin, Lichtenberg, Germany; 3 Universitätsmedizin Berlin, Institute for Laboratory Medicine and Pathobiochemistry, Charité Campus Mitte, Germany

## Abstract

**Background:**

Contrast echocardiography is a precise tool for the non-invasive assessment of myocardial function and perfusion. Side effects of contrast echocardiography resulting from contrast-agent induced myocardial micro-lesions have been found in animals. The goal of this study is to measure markers of myocardial necrosis, inflammation and oxidative stress in humans to evaluate potential side-effects of contrast echocardiography.

**Methods:**

20 patients who underwent contrast echocardiography with Optison as the contrast medium were investigated. To evaluate myocardial micro-necrosis, inflammation and oxidative stress, cardiac troponin I (cTnI), tumor necrosis factor-α (TNF-α), interleukin (IL)-6, -8 and thiobarbituric acid reactive substances (TBARS) were measured at baseline and at 2, 4, 8 and 24 hours after contrast echocardiography.

**Results:**

At baseline, 50% of the patients had cTnI and TBARS values outside the reference range. TNF-α, IL-6, IL-8 levels were within the reference range. Patients with cTnI above the RR clustered to significantly higher levels of TNF-α and IL-6. After contrast echocardiography, no statistically significant increase of cTnI, cytokines and TBARS was found. However, for nearly 50% of the patients, the intra-individual cTnI kinetics crossed the critical difference (threefold of methodical variation) which indicates a marker increase. This was neither predicted by the baseline levels of the cytokines nor the markers of oxidative stress.

**Conclusion:**

There are no clinically relevant increases in serum markers for micro-necrosis, inflammation and oxidative stress in humans after contrast echocardiography. Future studies have to address whether cTnI increase in some patients represent a subset with increased risk for side effects after contrast echocardiography.

## Background

Contrast echocardiography is a precise and study-proven tool for the non-invasive assessment of left ventricular systolic function, myocardial perfusion, endocardial border detection and coronary flow reserve [1,2].

Myocardial trauma, e.g. occlusion of a coronary artery during angioplasty, can potentially stimulate inflammatory processes with subsequent activation and infiltration of inflammatory cells in the myocardium. This can induce the release of cytokines and reactive oxygen species [3]. Microbubbles in their use as ultrasound contrast agents, which are destroyed by ultrasound, potentially also pose a risk for myocardial micro-lesions. This was demonstrated by *in vitro *and *in vivo *studies [4-7]. Therefore, in addition to markers of myocardial necrosis (cTnI), the measurement of markers for inflammation (TNF-α, IL-6, IL-8) and oxidative stress (TBARS) in peripheral blood is an appropriate tool to evaluate myocardial injury in humans undergoing contrast echocardiography.

These markers are not specific for myocardial damage but the degree of oxidative stress is correlated to the extent and progression of myocardial damage. [8-11]. It has been demonstrated in previous studies that levels of thiobarbituric acid reactive substances (TBARS) are elevated in patients with cardiovascular disease [12]. These patients might have a higher individual risk to develop side-effects from contrast myocardial echocardiography.

In a former study, we analyzed markers for myocardial necrosis (myoglobin, cardiac troponin I, creatine kinase MB activity and mass) in patients undergoing contrast echocardiography. None of the markers crossed the *cut-offs *generally accepted to indicate myocardial necrosis [13]. Possibly, one reason to these negative findings is that the *cut-off *values deducted from ROC-curves which were used at that time were too high for the detection of micro-lesions after contrast echocardiography. Based on strongly improved assay performance, it is now generally agreed that the elevation of cTnI without crossing the former *cut-off *is indicative of myocardial micro-necrosis [14]. Consequently, myocardial infarction without clinical symptoms – also termed "minor myocardial infarction" – is now re-defined as a troponin increase above the 99% percentile of the reference population or a troponin increase of more than the level that can be measured with an imprecision of = 10%, respectively [15-17].

Furthermore, there is increasing evidence that a low level troponin increases are pathologically relevant. Specifically, cTnI values below the ROC cut-off are clearly associated with mild and subclinical myocardial damage [18].

In this study, we performed additional measurements on the serum samples of the former study. cTnI concentrations were measured at baseline and at 2, 4, 8 and 24 hours after contrast echocardiography. Furthermore, we measured the markers of inflammation and oxidative stress (TNF-α, IL-6, IL-8 and TBARS).

## Materials and methods

20 consecutive patients with a clinical indication for contrast echocardiography were included in this study. The indications for contrast echocardiography are listed in table 1. The patients were clinically stable, older than 18 years of age and had signed an informed consent. Exclusion criteria included acute coronary syndrome, hemodynamic instability and cardiogenic shock. The study protocol was approved by the ethics committee of the Charité University Hospital. The venous blood sampling was performed immediately before (baseline) and 2, 4, 8 and 24 hours after contrast echocardiography. Due to limited availability of the plasma samples, in only 16 of the 20 patients all cytokines (TNF-α, IL-6, IL-8) could be measured.

**Table 1:** Baseline characteristics of the patients and indication for contrast echocardiography

**Diagnosis**	**n**	**Contrast imaging indication**
**Ischemic**	**n = 6**	
Coronary heart disease	6	Endocardial border detection / myocardial perfusion
**Non-ischemic**	**n = 14**	
Dilated cardiomyopathy	6	Endocardial border detection
Aortic valve replacement	2	Doppler flow enhancement / Endocardial border detection
Myocarditis	1	Endocardial border detection
Chronic obstructive lung disease	1	Endocardial border detection
Hypertensive heart disease	2	Endocardial border detection
Hypertrophic obstructive cardiomyopathy	2	Endocardial border detection / Doppler flow enhancement
**Total**	**20**	

The critical difference indicating a marker increase after echocardiography was calculated based on the data for the *within-run *variation. There is general agreement that two values measured *within-run *are significantly different (p < 0.05) if the difference between the values is greater than the three-fold of the *within-run *variation of the method.

Contrast echocardiography was performed with the Vivid Five System (GE Vingmed Ultrasound, Horten, Norway). Optison (perfluoro-propane-filled albumin microspheres, Mallinckrodt, St. Louis, Mo, USA) was used as the contrast medium. 20 ml of contrast microbubble solution (3 ml Optison and 17 ml 0.9% saline solution) were infused manually over three minutes into a peripheral vein followed by a saline flush. Contrast echo was performed 5 minutes after the beginning of the application of the contrast solution.

Cardiac troponin I was measured with the heterogeneous immunoassay module cardiac troponin-I flex™ reagent cartridge for Dimension (Date Behring, Marburg, Germany). For this assay, the lowest limit of detection (LoD) is 0.04 μg/l. The 99%-percentile of the reference group is 0.07 μg/l. In the range below the ROC cut off (0.6 μg/l), the analytical variation is 10% at a cTnI level of 0.24 μg/l and nearly 20 % at the 99 % percentile [14].

For TNF-α, IL-6 and IL-8, IMMULITE immunoassays obtained from DPC Biermann GmbH (Bad Nauheim, Germany) were used. According to the manufacturer information, reference ranges (95% percentile) and their analytical variation for TNF-α, IL-6 and IL-8 are 12% at 8.1 ng/l, 7% at 9.7 ng/l and 3.5% at 62 ng/l respectively. The LoD and the analytical variation at this level are 18% at 4 ng/l; 10% at 2 ng/l and 6% at 5 ng/l.

TBARS was measured according to TAKEDA et al. [19]. To avoid unspecific TBARS changes caused by *in vitro *oxidation and to guarantee high precision in the analytical procedures, adequate conditions for sampling, storage and measurement were ascertained in pre-study experiments [20]. Therefore, samples for TBARS were immediately frozen after sampling, stored in liquid nitrogen until measurement and analysed in parallel for high precision. Under these circumstances, the *within-run *variation was 3.8 %. There is no standardized test for TBARS, so that measurements have to be compared with the laboratory specific reference range. Based on former investigations, the reference group of 20 age-matched healthy controls had a mean value of 3.82 ± 0.51 μmol/l (min 2.62 – max 5.06 μmol/l). The 95%-percentile of the reference group is 4.79 μmol/l.

### Statistical Analysis

Data are expressed as mean ± SD. Statistical analysis was performed with the SPSS 12 software package (SPSS; Chicago, Ill, USA). Using bootstrapping simulation, the U-test of Mann-Whitney for group comparison and the Wilcoxon test for comparison of paired values (pre-study value vs. maximum value following contrast echocardiography) were used. In the figures, the measurements below the LoD are displayed by the values that correspond to half of the LoD.

## Results

### A) Marker levels at baseline

#### cTnI

**Figure 1:**
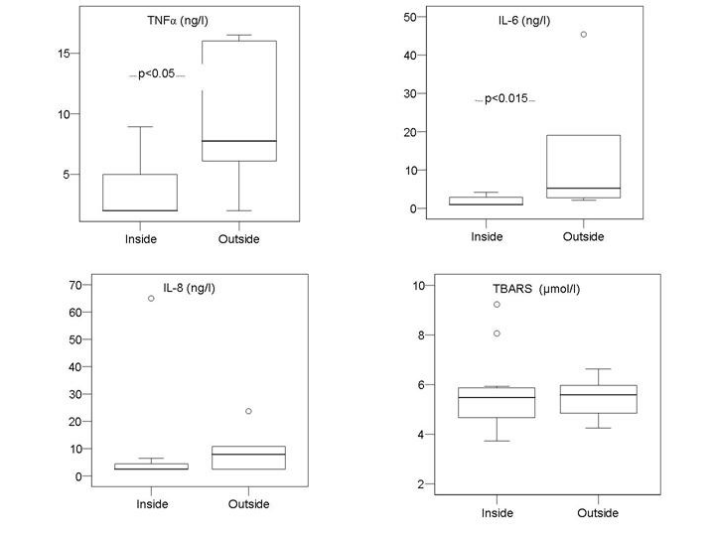
Boxplot analysis of the baseline concentration of TNF-α, IL-6, IL-8 and TBARS in the plasma of patients with plasma cTnI inside or outside of the reference range. Despite the significant differences in the mean values of TNF-α and IL-6 at baseline, the intra-individual changes were not statistically significant.

**Figure 2:**
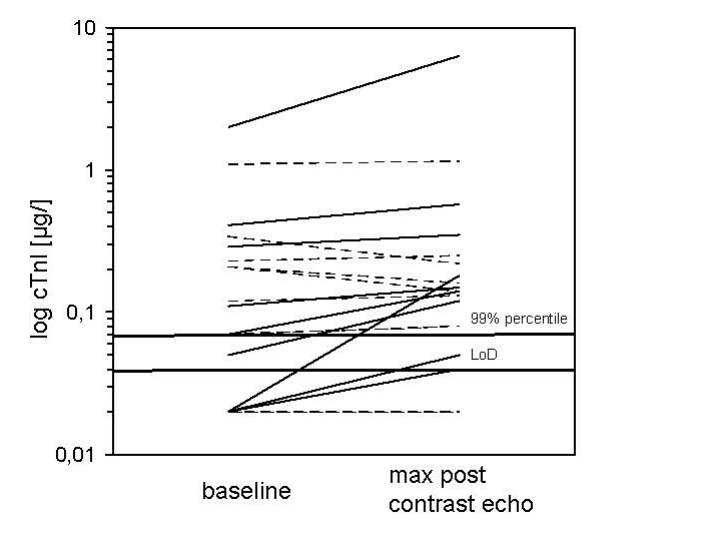
Increase of cTnI after contrast echocardiography: Baseline value and the maximum value within 24 hours after contrast echocardiography. The dotted lines indicate patients with no significant increase. The black lines represent patients with an increase greater than the three-fold of the *within-run *variation of the method.

7 of the 20 patients had baseline cTnI levels below the LoD (0.04 ug/l). 3 patients had a cTnI level between the LoD and the 99%-percentile of the reference population (≤ 0.07μg/l). Altogether, 10 patients were within the reference range. The other 10 patients had baseline cTnI levels above the reference range: 8 patients with values between the 99%-percentile and the ROC *cut-off *of 0.6 μg/l and 2 patients with values which were even above the ROC *cut-off*. (see table 2, figure 1). As shown in figure 2, one patient had a much higher baseline cTnI level (1.99μg/l) than all the others. The patient was diagnosed with chronic dilated cardiomyopathy and intermittent ventricular bigeminus and couplets. In contrast to elevated levels of cTnI and TBARS, he had normal levels of the other markers at baseline.

**Table 2:** Classification of patients according to baseline levels of cTnI, TNF-α, IL-6, IL-8 and TBARS with respect to inside or outside the reference range

	**Within reference range**	**Outside reference range**
cTnI (n = 20)	10	10
TNF-α (n = 16)	11	3
IL-6 (n = 16)	14	2
IL-8 (n = 16)	16	0
TBARS (n = 20)	6	14

#### Cytokines

For TNF-α (reference range ≤ 8.1 ng/l), 3 of the 16 patients had baseline values above the reference range. Two patients belonged to the group with cTnI in the reference range, one to the group with cTnI above the reference range.

However, comparing all baseline TNF-α levels to the cTnI levels (inside or outside the reference range), TNF-α was significantly lower in the patient group with cTnI in the reference range (3.8 ± 2.6 vs. 9.4 ± 5.7 ng/l ; 95 % confidence interval of the p value 0.030 – 0.037) than the patients with cTnI above the reference range. The correlation of cTn-I and TNF-α is also documented by the regression analysis (r = 0.57; p = 0.043).

For IL-6 (reference range of ≤ 9.7 ng/l), two patients both from the cTnI group outside of the reference range had IL-6 values outside of the reference range. In analogy to the TNF-α levels, there was a significantly higher IL-6 level in the patient group outside of the cTnI reference range compared with that inside (vs. 2.0 ± 1.0 vs.13.3 ± 16.9 ng/l; 95 % confidence interval of the P value 0.009 to 0.014). The linear regression of baseline IL-6 and cTnI was r = 0.68 (p = 0.01). A significant correlation of baseline TNF-α and IL-6 could be calculated (r = 0.58; p < 0.05).

For IL-8, the baseline values of all patients were all within the reference range. There was no significant difference in IL-8 values between the patient groups inside and outside of the cTnI reference interval (13.8 ± 22.6 vs. 10.0 ± 7.2 ng/l).

#### Oxidative stress

Based on the calculated reference range (95%-percentile) the TBARS level of 6 of the patients was inside the reference range, while in 14 patients it was outside of the reference range at baseline. TBARS levels were significantly higher in the patient group compared to the healthy controls: mean 5.82 ± 1.30 μmol/l (min 4.18 μmol/l, max 9.23 μmol/l, 95 % confidence interval of the P value: 0.001 to 0.0001). However, no significantly different TBARS levels (5.98 ± 1.65 vs 5.61+0.81 μmol/l) were found in the cTnI groups inside or outside the reference range. There was no significant correlation of baseline TBARS to cTnI, TNF-α and IL-6, however, the TBARS correlated significantly to the baseline IL-8 (r = 0.82; p < 0.01).

Overall, if patients were classified based on their underlying heart disease in "ischemic" (n = 6) and "non-ischemic" (n = 14), no significantly different pre-study levels for all parameters are present.

### B) Marker levels after contrast echocardiography

#### cTnI

**Figure 3:**
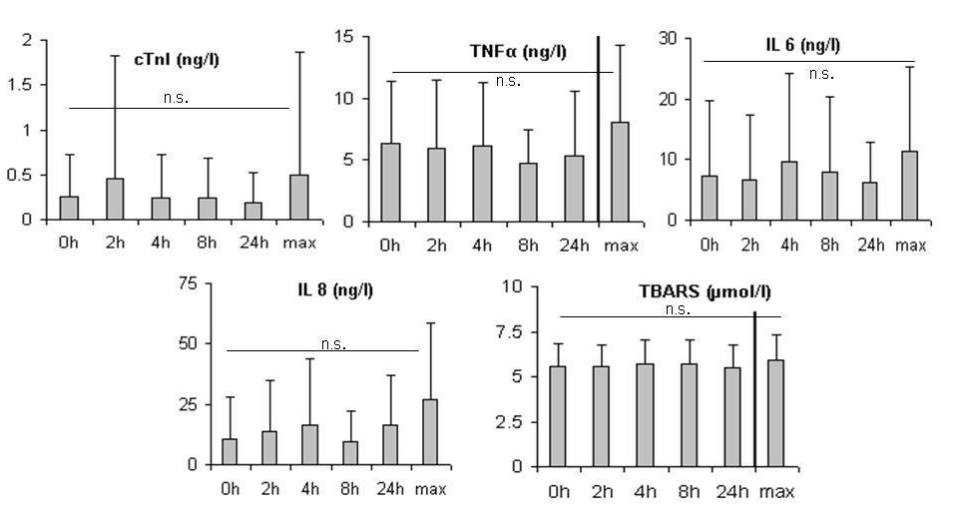
Time kinetics of cTnI, TNF-α, IL-6, IL-8 and TBARS levels at baseline and at 2, 4, 8 24 hours and maximum value after contrast echocardiography.

As shown in figure 1 the mean cTnI levels were 0.26 ± 0.47 ng/l (baseline), 0.47 ± 1.37 ng/l (2 hours), 0.24 ± 0.48 ng/l (4 hours), 0.24 ± 0.45 ng/l (8 hours), 0.18 ± 0.33 (24 hours). Based on the definition of marker increase as described in material and methods, the intra-individual kinetics showed a cTnI increase for 9 patients after contrast echocardiography at one or more time points compared to baseline (figure 2). The strongest increase (to 6.35 μg/l) was found for the patient with the highest baseline cTnI. This high value was only found 2 hours after contrast echocardiography and returned to baseline thereafter. This patient had no significant changes of the cytokine and TBARS levels. Despite a cTnI increase in some patients, statistical analysis using the Wilcoxon test for comparison of paired values did not reveal a significant increase for the whole group of patients (figure 3).

There was no predictive power of baseline cTnI levels for an increase after contrast echocardiography. In the group with baseline cTnI within the reference range, we found an increase based on our definition in 5 of the 10 patients. In the group with baseline cTnI above the reference range, the increase was seen in 4 of the 10 patients.

Neither the etiology of the heart disease (ischemic and non-ischemic) nor the baseline cTnI level predicted an increase in cTnI.

#### TNF-α and Cytokines

In the case of TNF-α and cytokines, the critical difference was crossed 24 hours after contrast echocardiography for TNF-α in 3, for IL-6 in 6 and for IL-8 in 7 of the analyzed patients. The marker increase was neither related to the baseline cTnI nor to the ischemic or non-ischemic background. The correlation of cytokine and cTnI increase was not statistically significant.

#### Oxidative stress

The oxidative stress marker TBARS exceeded the critical difference after contrast echocardiography in 7 patients. The increase of TBARS was neither related to the baseline cTnI nor to the ischemic or non-ischemic background of the underlying myocardial pathology of the patient.

## Discussion

This study was designed to investigate safety of contrast echocardiography in humans. By determination of serum markers for myocardial necrosis, inflammation and oxidative stress, no statistically significant side-effects could be detected. In the most of the patients, the baseline marker levels were within or near to the reference range and there were no sustained increases 24 hours after contrast echocardiography.

As marker for myocardial necrosis, cTnI was chosen because the analytical sensitivity of the test has improved and it is now accepted as an indicator of myocardial micro-necrosis. Furthermore, there is increasing evidence that cTnI can indicate myocardial damage that is not associated with necrosis [21-24].

To examine inflammatory activation after contrast echocardiography, TNF-α and IL-6 and IL-8 were analyzed. These markers are not specific for a myocardial inflammatory response, but a marker increase with temporal association to contrast echocardiography more likely results from the proposed effects of the contrast agent on the heart than from systemic inflammation by other causes [25].

TBARS, a metabolite and end product of lipid peroxidation, is a more static and global parameter. TBARS is, despite the lack in standardization, currently the most frequently used marker to quantify oxidative stress in clinical studies. It was measured in several studies in patients with heart diseases and increased oxidative stress [12,26]. The patients in our study had significantly higher baseline TBARS levels than the control group of age-matched healthy subjects. This is in agreement with the data demonstrating that the induction of oxidative stress is a sequelae in the pathogenesis of heart diseases.

Our results after contrast echocardiograph in humans are be in agreement with a recently published study. A new second-generation myocardial contrast agent (LK565) has been evaluated in healthy individuals undergoing contrast echocardiography. Neither an increase in TNF-α secretion, nor antibody development and activation of leukocyte surface markers was induced by the procedure [27]. However, these results found in healthy subjects cannot be simply transferred to studies such ours since patients with heart disease could develop side-effects which are more pronounced in quality and quantity.

Consequently and in critical view of our study results, we hesitate to negate a potential myocardial damage after contrast echocardiography. An intra-individual increase of cTnI above the pre-determined limit was seen in nearly half of the patients. Furthermore, IL-6 and IL-8 increased after contrast echocardiography based on the pre-defined limit in nearly half of the patients analysed for cytokines. The increases of cTnI, IL-6 and IL-8 were not correlated to a particular group of patients. Further larger studies have to clarify whether subgroups of patients are prone to myocardial damage after contrast echocardiography. In present study, we cannot exclude that the findings in some of our patients resulted from such a subset enrolled in our patient cohort. Due to the sample size we were not able to clearly define subgroups. In our view, patients with cTnI or TBARS above the reference range at baseline could form such a subgroup more susceptible to side- effects.

Possibly, the one patient suffering from dilated cardiomyopathy and ventricular arrhythmia who had the highest baseline and largest increase in cTnI levels after contrast echocardiography could belong to such a subgroup. High troponin levels are found in patients with stable heart failure without an indication for myocardial ischemia [20-22,28]. Furthermore, patients with tachycardia can have persistently high troponin levels [29] which might explain the elevated baseline level of cTnI in our patient affected by intermittent ventricular bigeminus and ventricular couplets. However, the cTnI increase after contrast echocardiography in this patient was neither reflected by a rise in cytokines nor in oxidative stress and was not accompanied by clinical signs and symptoms of myocardial ischemia. Therefore, it is unclear whether this cTnI increase is linked to the contrast echocardiography procedure alone or if it primarily reflects the underlying heart disease.

We have found a significant correlation of the baseline levels of cTnI, TNF-α and IL6. This is in accordance with the findings of [30], who found an association of troponin and cytokine levels in heart disease.

Limitations of this study include the low number of patients, the lack of a control group and the use of just one contrast agent. Increasing the number of patients studied would facilitate the identification of patients who are more sensitive to the side-effects of contrast echocardiography. Controls in future studies would have to receive contrast agent only without echocardiography. We cannot draw comparisons between the effects of different contrast agents because only Optison was used in this study.

In summary, there is no conclusive evidence for the induction of myocardial micro-necrosis, inflammation and oxidative stress by contrast echocardiography. The baseline cTnI levels do not correlate to the oxidative stress and inflammation markers within 24 hours after contrast echocardiography. In some of the patients however, cTnI levels increase slightly after contrast echocardiography. These patients may belong to a subgroup more prone to side effects of contrast echocardiography than others. Hence, this study – although limited in size – suggests that contrast echocardiography does not induce oxidative stress and inflammation.

## Abbreviations

TNF-α Tumor necrosis factor alpha

TBARS thiobarbituric acid reactive substances

IL Interleukin

ROC Reviever operating characteristic curve

cTnI cardiac Troponin I

LoD lowest limit of detection

MI myocardial infarction

## Authors' contributions

FK, IS and ACB have designed and performed the study and have written the manuscript. IS, FK, RZ and ACB have performed the laboratory measurements. TW, SE, SS and GB have participated in the study design and coordination. All authors read and approved the final manuscript.
